# Synthesis of 2,3,6,7-tetrabromoanthracene

**DOI:** 10.3762/bjoc.4.41

**Published:** 2008-11-10

**Authors:** Christian Schäfer, Friederike Herrmann, Jochen Mattay

**Affiliations:** 1 Universität Bielefeld, Organische Chemie I, Universitätsstr. 25, 33615 Bielefeld, Germany

**Keywords:** anthracene, arenes, cyclizations, polycycles, ring closure, 2,3,6,7-tetrabromoanthracene

## Abstract

The first synthesis of 2,3,6,7-tetrabromoanthracene is presented, starting from benzene in a straightforward four step synthesis.

## Introduction

Anthracene and its derivatives are long known polycyclic aromatic compounds showing a high potential for use in materials science (e.g. fluorescence probing, photochromic systems, electroluminescence) and several reviews have been published so far [1–3]. Anthracenes may be readily functionalized in positions 9 and 10 due to their exceptional reactivity. The outer rings, however, can not be functionalized easily. There are some anthracene compounds available with one or two substituents at the 1- or 2-positions (such as 1- or 2-aminoanthracene), but to make the 2-, 3-, 6- and 7-position chemically available for further reactions, great effort is necessary. The only exception is maybe 2,3,6,7-tetramethylanthracene, which was first reported in 1931 by Morgan and Coulson [4].

In 1988, Lin and Chou presented the first synthesis of 2,3-dibromoanthracene [5], using a Diels-Alder reaction as the key step in synthesis. Twelve years later, Bowles and Anthony published an alternative synthesis for the same compound using a Bergman cyclization [6]. There are several other publications involving brominated anthracenes [7–12], mainly using anthraquinones for the bromination at an outer position, but a synthesis of 2,3,6,7-tetrabromoanthracene is missing. In this paper, we report a convenient four step synthesis of 2,3,6,7-tetrabromoanthracene via a twofold Bergman cyclization. This tetrabromide constitutes an excellent precursor for 2,3,6,7-tetrasubstituted anthracenes due to the bromine groups and it is also a candidate precursor to 2,3,6,7,9,10-hexabromoanthracene because of the high reactivity of the 9,10-positions. In addition, new materials may also be accessible, such as 2,3,6,7-tetradehydroanthracene (for constructing polycyclic aromatic hydrocarbons [13–14]) and 2,6,9,10-tetracyanoanthracene (which has been used as sensitizer for organic photoconductors [15]).

## Results and Discussion

**Scheme 1:**
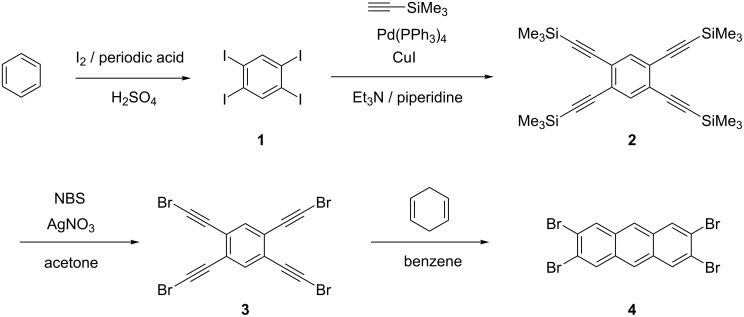
Synthesis of 2,3,6,7-tetrabromoanthracene (**4).**

In Scheme 1, the reaction pathway is shown, starting from benzene, which is iodinated four times to give **1** in 71% yield by the method of Mattern [16]. The next step is a fourfold Sonogashira-Hagihara coupling reaction with trimethylsilylacetylene. We slightly modified the procedure reported by Vollhardt et al. [17] and used tetrakis(triphenylphosphine)palladium(0) as catalyst, obtaining **2** in 92% yield. In the following step, the trimethylsilyl groups were substituted by bromine atoms, analogous to the procedure reported by Bowles and Anthony [6]. The reaction yielded nearly 100% of **3** as a slightly yellow solid after flash chromatography with dichloromethane on silica gel. Although we succeeded in characterizing **3** by ^1^H NMR, the neat product is not stable. In fact, dry **3** is explosive and it should be used directly for the final step of the synthesis. In a first attempt, 1,2-dichlorobenzene was used as high boiling solvent and γ-terpinene as hydrogen donor leading to 2,3,6,7-tetrabromoanthracene (**4**), which was isolated from possible byproducts by column chromatography and recrystallized from ethanol. However, even after this work-up, the solvent was still present according to NMR analysis. In a second attempt, the reaction took place in benzene at 180–200 °C in a steel bomb, using 1,4-cyclohexadiene as hydrogen donor. In this case, the structure and the purity of **4** were proven by NMR and high resolution mass spectrometry.

**Figure 1:**
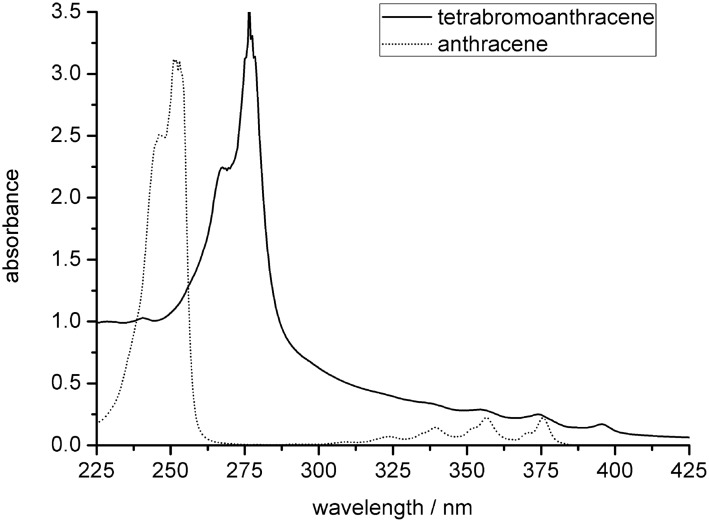
UV-spectra of **4** and commercially available anthracene in cyclohexane (9 × 10^−5^ mol/L).

The UV/VIS-spectra of 2,3,6,7-tetrabromoanthracene and anthracene in cyclohexane are shown in Figure 1. At first glance, both spectra resemble each other; however, all absorption bands are bathochromically shifted (~25 nm difference) and the typical anthracene absorption is broadened for **4** due to the heavy atom effects of the bromo substituents. Both spectra are cut off at 220 nm. Below this wavelength, the absorbance for both substances decreases slightly until the solvent (cyclohexane) starts to absorb.

In conclusion, we developed a straightforward four step synthesis of 2,3,6,7-tetrabromoanthracene, starting from benzene by using a double Bergman cyclization as key step.

## Experimental

The NMR spectra were recorded on a 500 MHz NMR spectrometer (Bruker, DRX 500). GC/MS spectra were measured on the Shimadzu GC17A/GCMS-QP5050 mass spectrometer equipped with a standard EI source. High resolution EI mass spectra were recorded using an Autospec X magnetic sector mass spectrometer with EBE geometry (Vacuum Generators, Manchester, UK) equipped with a standard EI source. Samples were introduced by push rod aluminium crucibles. Ions were accelerated by 8 kV in EI mode. Benzene was dried over molecular sieve 4 Å for at least 24 h prior to use. All other solvents and chemicals were used without further purification. Melting points were measured on the B-540 (Büchi) and were not corrected.

### 1,2,4,5-tetraiodobenzene (**1**)

Periodic acid (5.21 g, 23 mmol) was dissolved in 100 mL conc. sulfuric acid and iodine (17.40 g, 68 mmol) was added. After 30 min, the flask was cooled in an ice bath and benzene (3.6 mL, 40 mmol) was added slowly. The reaction was stirred overnight, allowed to reach ambient temperature and added to ice. The precipitated product was filtered and recrystallized from 2-methoxyethanol and dried under vacuum; yield: 16.52 g (28.4 mmol; 71%); mp 255 °C (Lit. [16] 252–255 °C); ^1^H NMR (DMSO-*d*_6_): δ [ppm] = 8.31 (ArH); ^13^C NMR (DMSO-*d*_6_, +80 °C): δ [ppm] = 109.0 (C_Ar_I), 146.8 (C_Ar_H); GC/MS (EI): *m/z* (%) = 455 (27) [M–I]^+^, 328 (65) [M–2I]^+^, 291 (84), 201 (100) [M–3I]^+^, 200 (41), 127 (29) [I]^+^. The M^+^-signal could not be detected; the ^13^C NMR was measured at 80 °C due to low solubility.

### 1,2,4,5-tetrakis-trimethylsilylethynyl-benzene (**2**)

A solution of tetraiodobenzene (**1**) (4.90 g, 8.4 mmol) in 150 mL of Et_3_N and 30 mL of piperidine was degassed with argon (three pump-freeze-thaw cycles) and trimethylsilylacetylene (6.4 mL, 45 mmol), Pd(PPh_3_)_4_ (590 mg, 0.51 mmol) and CuI (65 mg, 0.34 mmol) were added. After 30 min, the reaction was heated to 80 °C for 2 h and stirred overnight at room temperature. Cyclohexane was added and the whole mixture was washed with diluted hydrochloric acid until the water layer stayed acidic. The organic layer was washed with brine, dried over MgSO_4_ and the solvent was evaporated. After column chromatography on silica gel (eluent: cyclohexane), the product was dried under vacuum; yield: 3.59 g (7.8 mmol; 92%); *R_f_* 0.14 (cyclohexane); mp 163–164 °C (Lit. [17] 163.5–164 °C); ^1^H NMR (C_6_D_6_): δ [ppm] = 0.24 (s, 36 H, CH_3_), 7.58 (s, 2 H, ArH); ^13^C NMR (C_6_D_6_): δ [ppm] = 0.1 (-Si(CH_3_)_3_), 101.0 (C_ethinyl_), 102.9 (C_ethinyl_), 126.0 (C_Ar_), 136.5 (C_Ar_); GC/MS (EI): *m/z* (%) = 464 (26) [mainly M(1× ^30^Si) and M(2× ^29^Si)], 463 (48) [M(1× ^29^Si) and M(1× ^13^C)], 462 (100) [M_mono_], 448 (19), 447 (39) [M–CH_3_], 389 (5) [M–SiMe_3_], 360 (9), 359 (33), 216 (8).

### 1,2,4,5-tetrakis-bromoethynyl-benzene (**3**)

1,2,4,5-tetrakis(trimethylsilylethynyl)benzene (**3**) (830 mg, 1.79 mmol) was suspended in 30 mL acetone under argon and NBS (1.60 g, 9.0 mmol) and AgNO_3_ (0.30 g, 1.77 mmol) were added. The suspension was stirred for 18 h at ambient temperature, the solvent evaporated and the residue filtered on a pad of silica gel or a small column (eluent: dichloromethane). After evaporation of the solvent, the product was used for the next reaction immediately due to fast decomposition; yield: ~quantitatively; *R_f_* 0.76 (dichloromethane); ^1^H NMR (CDCl_3_): δ [ppm] = 7.47 (s, 2 H, ArH). No ^13^C NMR was recorded due to fast decomposition; the ^1^H NMR showed no TMS groups indicating a complete substitution. CAUTION: The dry substance is explosive.

### 2,3,6,7-tetrabromoanthracene (**4**)

1,2,4,5-tetrakis(bromoethynyl)benzene (**3**) (877 mg, 1.79 mmol) and 50 mL of dry benzene were placed in a steel bomb and degassed with argon for 15 min. 1,4-cyclohexadiene (5 g, 62 mmol) was added and the mixture was degassed with argon for another 10 min. The steel bomb was closed and heated to 180–200 °C for 2.5 h. After the reaction cooled off, the solvent was evaporated under vacuum and the residue filtered on silica gel (eluent: dichloromethane) and recrystallized from ethanol; yield: 512 mg (1.04 mmol, 58%), *R_f_* 0.79 (dichloromethane); *R_f_* 0.27 (cyclohexane); mp >220 °C (decomposition); ^1^H NMR (CDCl_3_): δ [ppm] = 8.20 (s, 2 H, ArH), 8.30 (s, 4 H, ArH); ^13^C NMR (CDCl_3_): δ [ppm] = 122.8 (C_Ar,q_), 124.8 (C_Ar_H), 131.2 (C_Ar,q_), 132.2 (C_Ar_H); UV spectrum: λ_max_(cyclohexane)/nm (ε/dm^3^ mol^−1^ cm^−1^) 395 (1880), 374 (2770), 354 (3210), 277 (36500), 267 (24900); HRMS (EI): *m/z* calcd for C_14_H_6_Br_4_: 489.72030; found: 489.71920.
